# Characterizing circadian rest–activity rhythm patterns across Alzheimer's disease continuum in Down syndrome

**DOI:** 10.1002/alz.71409

**Published:** 2026-04-29

**Authors:** Sandra Giménez, Lídia Vaqué‐Alcázar, Susana Clos, Bessy Benejam, Maria Carmona‐Iragui, Lucía Maure‐Blesa, Laura Videla, Nuole Zhu, Miren Altuna, Javier Arranz, Isabel Barroeta, Íñigo Rodríguez‐Baz, Alexandre Bejanin, Ana Bueno, Susana Fernandez, Laura del Hoyo Soriano, Lucia Pertierra, Daniel Alcolea, Bruce Miller, Lea T. Grinberg, Joaquin Ruiz, Christos Panagiotis Lisgaras, Hau‐Tieng Wu, Alberto Lleó, Ricardo S. Osorio, Esther M. Blessing, Juan Fortea

**Affiliations:** ^1^ Multidisciplinary Sleep Unit Respiratory Department Hospital de la Santa Creu i Sant Pau Biomedical Research Institute Sant Pau (IIB SANT PAU) Barcelona Spain; ^2^ Sant Pau Memory Unit Department of Neurology Hospital de la Santa Creu i Sant Pau Biomedical Research Institute Sant Pau (IIB SANT PAU) Universitat Autònoma de Barcelona Barcelona Spain; ^3^ Center of Biomedical Investigation Network for Neurodegenerative Diseases (CIBERNED) Madrid Spain; ^4^ Global Brain Health Institute University of California San Francisco California USA; ^5^ Department of Medicine Faculty of Medicine and Health Sciences Institute of Neurosciences University of Barcelona Institut d'Investigacions Biomèdiques August Pi i Sunyer (IDIBAPS) Barcelona Spain; ^6^ Barcelona Down Medical Center Fundació Catalana de Síndrome de Down Barcelona Spain; ^7^ Center for Research and Memory Clinic CITA‐Alzheimer Foundation Donostia‐San Sebastián Spain; ^8^ Debabarrena Integrated Health Organization Osakidetza Basque Health Service Mendaro Spain; ^9^ Department of Neurology Hospital de Araba Bioaraba Health Research Institute Araba University Hospital‐Txagorritxu Vitoria‐Gasteiz Spain; ^10^ Cognitive Neurology, Neuropsychology and Neuropsychiatry Unit Fleni Memory Clinic Buenos Aires Argentina; ^11^ Edward and Pearl Fein Memory and Aging Center University of California San Francisco California USA; ^12^ Departments of Laboratory Medicine and Pathology and Neurosciences Mayo Clinic Jacksonville Florida USA; ^13^ Department of Psychiatry New York University Langone Health New York City New York USA; ^14^ The Nathan S. Kline Institute for Psychiatric Research New York State Office of Mental Health Orangeburg New York USA; ^15^ Department of Mathematics at the Courant Institute of Mathematical Sciences New York University New York City New York USA

**Keywords:** actigraphy, aging, Alzheimer's disease, circadian rest–activity rhythm patterns, Down syndrome, sleep

## Abstract

**INTRODUCTION:**

Sleep and circadian rest–activity rhythm (RAR) disruption may bidirectionally relate to Alzheimer's disease (AD). Down syndrome (DS), the most common genetic cause of AD, presents sleep disorders, yet RAR patterns across the DS‐associated AD continuum remain uncharacterized.

**METHODS:**

We analyzed 7‐day wrist actigraphy in 140 adults with DS (108 asymptomatic; 32 AD dementia) and 41 unimpaired controls. General linear models, adjusted for age, sex, sleep efficiency, and obstructive sleep apnea (OSA) severity, tested group differences, with interaction terms included to evaluate group‐specific associations.

**RESULTS:**

DS showed lower relative amplitude and higher nocturnal activity, already in asymptomatic individuals. Rhythm strength declined further with AD progression, while regularity and phase timing remained preserved until dementia. Findings were independent of sleep duration and OSA.

**DISCUSSION:**

Adults with DS showed early RAR disturbance that progressed across the AD continuum, paralleling sporadic AD. Circadian RAR features may be scalable biomarkers of AD progression.

## Highlights

- In DS, circadian RAR is already disrupted prior to dementia onset, with reduced amplitude and increased nocturnal activity that worsen across the AD spectrum.
- Circadian RAR is primarily driven by loss of robustness of the rhythm rather than changes in phase–phase measures of daily regularity or rhythm timing.
- Circadian RAR alterations persist after adjustment for age and nocturnal sleep disturbances, indicating intrinsic circadian dysregulation.
- Actigraphy‐derived circadian measures may provide scalable, non‐invasive markers of early AD vulnerability in DS populations.

## 1 BACKGROUND

Down syndrome (DS) is the most common genetic form of Alzheimer's disease (AD) and the leading genetic cause of intellectual disability.^1^ Triplication of chromosome 21 includes the amyloid precursor protein (APP) gene, leading to lifelong amyloid beta (Aβ) overproduction and the nearly universal development of AD pathology by age 40. Consequently, over 90% of adults with DS eventually develop dementia, and AD is the primary cause of death after age 35.^2^, ^3^, ^4^ Importantly, AD biomarker trajectories in DS closely mirror those observed in sporadic and autosomal‐dominant AD (ADAD), positioning DS as a uniquely informative human genetic model for studying AD pathophysiology and progression.^3^, ^5^, ^6^, ^7^

Sleep fragmentation and sleep disorders, particularly obstructive sleep apnea (OSA), are highly prevalent in adults with DS and tend to worsen with age.^8^, ^9^, ^10^ We recently demonstrated that AD pathology in DS exacerbates these sleep disturbances, with increasing OSA severity and decreasing rapid eye movement (REM) sleep, suggesting that neurodegeneration amplifies age‐related sleep impairment.^11^

In the general population, growing evidence indicates a bidirectional relationship between sleep and circadian rhythm disruption and dementia risk.^12^, ^13^, ^14^, ^15^, ^16^ Clinically, AD is characterized by marked circadian sleep–wake disorganization with increased nighttime activity, reduced daytime alertness, and behavioral dysregulation – symptoms strongly associated with caregiver burden and institutionalization.^17^, ^18^, ^19^

The circadian system, governed by its principal pacemaker in the suprachiasmatic nucleus (SCN), synchronizes physiological and behavioral processes with the 24‐h light–dark cycle and associated environmental cues.^20^ Actigraphy‐derived rest–activity rhythm (RAR) measures offer a means of quantifying circadian rhythmicity by capturing daily activity and rest patterns in a patient's habitual home environment.^21^ Emerging neuropathological evidence indicates that the SCN is affected early in AD, with tau and glial‐related changes that may disrupt clock function and contribute to circadian symptoms early in the disease course.^22^ In euploid cohorts, RAR disturbances emerge in preclinical AD,^15^, ^23^ and reduced circadian amplitude and increased rest–activity fragmentation are associated with worse cognition, faster cognitive decline, and higher risk of mild cognitive impairment (MCI) and AD dementia.^19^, ^24^, ^25^, ^26^ These findings suggest that RAR disturbances may represent early and potentially modifiable markers of AD vulnerability.

Despite the high burden of sleep disturbances in DS and their worsening along the AD continuum, circadian rhythmicity in this population remains understudied. To date, only one small study has examined RAR in young adults with DS, finding earlier circadian disturbances compared to euploid controls.^27^ No prior work has characterized RAR measures across the full clinical AD spectrum in DS. Therefore, we aimed to provide the first comprehensive characterization of circadian RAR patterns in adults with DS across the AD continuum, in a comparatively large, deeply phenotyped cohort. We examined whether (i) circadian disruption was already detectable in asymptomatic individuals, (ii) RAR disturbance worsened along the AD continuum, (iii) these alterations persist independently of nocturnal sleep disturbances, such as OSA severity and sleep efficiency, and (iv) the age and sex influences on circadian RAR profiles in DS.

Characterizing RAR profiles from asymptomatic stages through dementia in DS may reveal early functional signatures of underlying AD pathology, improve risk stratification, and inform targeted interventions.

## 2 METHODS

### 2.1 Study design and participants

**RESEARCH IN CONTEXT**

1. **Systematic review**: We conducted a PubMed search for studies examining circadian RAR patterns in AD across sporadic and DS‐associated AD. Prior research in the euploid population reported a bidirectional relationship between circadian RAR disturbances and AD. No studies have investigated circadian RARs in adults with DS across the full AD clinical continuum.
2. **Interpretation**: Our findings demonstrate that adults with DS exhibit early loss of circadian rhythm robustness that worsens with AD progression, independently of age, SE, and OSA. This pattern mirrors that observed in sporadic AD, supporting circadian dysregulation as an early behavioral manifestation of AD‐related neurodegeneration in this genetically determined population.
3. **Future directions**: Longitudinal multimodal studies integrating actigraphy with biological circadian markers and AD biomarkers are needed to determine causal relationships and to evaluate circadian RAR measures as scalable markers and potential therapeutic targets across AD etiologies.

This single‐center cross‐sectional analytical study included adults with DS of both sexes recruited from the population‐based Down Alzheimer Barcelona Neuroimaging Initiative (DABNI), established by the Hospital de Sant Pau and the Catalan Foundation for Down Syndrome in Barcelona. DABNI is a multimodal AD biomarker study and longitudinal cohort nested within a population‐based screening program for neurological conditions in DS. The goal of DABNI is to advance interventions and clinical research in DS‐associated AD^7^ by comprehensively integrating clinical, cognitive, and multimodal biomarker data in adults with DS, starting at age 18. A control group of healthy controls (HCs), that is, cognitively unimpaired euploid adults, was drawn from the Sant Pau Initiative on Neurodegeneration (SPIN) cohort.^28^ All participants underwent comprehensive medical, neurological, neuropsychological, and sleep evaluations.

The study was approved by the Clinical Research Ethics Committee of Hospital de Sant Pau and conducted in accordance with the ethical principles outlined in the Declaration of Helsinki. Written informed consent was obtained from all participants or their legal guardians, and assent was obtained from participants with DS whenever possible prior to enrollment.

### 2.2 Clinical assessment

All participants underwent detailed medical, neurological, and neuropsychological evaluations. Global cognitive performance in adults with DS was assessed using the Spanish adaptation of the Cambridge Cognitive Examination for Older Adults with Down Syndrome (CAMCOG‐DS).^29^ The degree of intellectual disability (ID) was determined following the criteria of the *Diagnostic and Statistical Manual of Mental Disorders, Fifth Edition* (DSM‐5), supported by caregiver reports of best‐ever adaptive functioning and scores from the Spanish version of the Kaufman Brief Intelligence Test (KBIT).

As in prior DABNI studies,^3^, ^7^, ^11^ participants were clinically classified according to their stage along the AD continuum based on consensus between neurologists and neuropsychologists, who were masked to biomarker data, into three groups: (i) asymptomatic (aDS) for no clinical or neuropsychological suspicion of AD‐related cognitive decline; (ii) prodromal AD (pDS) for evidence of cognitive decline due to AD, but no significant impact on baseline activities of daily living; and (iii) AD dementia (dDS) if cognitive decline impaired daily activities.

Individuals with neurological disorders unrelated to AD (stroke, traumatic brain injury) or with new recent use (within 3 months) of psychoactive medications were excluded. The presence or absence of sleep‐related complaints was not used as an inclusion or exclusion criterion.

The HC group exhibited no cognitive complaints, scoring 0 on the Clinical Dementia Rating scale, and their neuropsychological evaluation was within the normal range for their respective normative groups regarding age and education. None of the participants in the HC group reported any neurological or psychiatric disorders or other major medical illnesses.

### 2.3 Actigraphy acquisition and processing

All participants wore an ActiGraph device (ActiGraph GT9X Link, ActiGraph, Pensacola, FL, USA) continuously on the non‐dominant wrist for seven consecutive days and completed concurrent sleep–wake logs documenting bedtimes, wake times, and daytime naps. For participants with DS, sleep logs were completed by caregivers, who were also trained to ensure device compliance and accurate reporting of daily sleep–wake behaviors. The nocturnal rest interval (time in bed) and the subsequent wake period (time out of bed) were defined based on caregiver‐completed sleep–wake logs and cross‐validated with actigraphy data. When sleep diaries were unavailable, bedtimes and wake times were imputed from available records or estimated by visual inspection of actograms. Raw accelerometry data were measured in the three axes (vertical, horizontal, and perpendicular), sampled at 30 Hz and aggregated into 60‐s epochs and visually inspected for recording quality and completeness. All collected data were downloaded using ActiLife software version 6.13.4 (ActiGraph LLC), and the activity counts were derived from the vector magnitude of the three axes. Any 24‐h periods with more than three continuous hours of missing data or recordings shorter than 36 continuous hours were excluded from the analyses.

### 2.4 Actigraphy‐derived parameters encompassed measures of circadian RAR and nocturnal sleep

Circadian RAR measures were analyzed using both non‐parametric and parametric approaches, which respectively assume or do not assume a sinusoidal pattern of the data. *Non‐parametric circadian RAR* measures were computed from actigraphy raw data using the R package nparACT (version 0.8) following established procedures.^30^, ^31^ The non‐parametric RAR measures estimated were as follows: (i) intradaily variability (IV), which quantifies the degree of fragmentation of the RAR within each 24‐h period, with values ranging from 0 to 2, where higher scores indicate more transitions between rest and activity states; (ii) the interdaily stability (IS), which measures the regularity of activity patterns across days, with values between 0 (random, irregular rhythm) and 1 (perfect day‐to‐day stability); (iii) M10, which is the mean activity across the 10 consecutive most‐active hours; (iv) L5, which is the mean activity across the five consecutive least‐active hours. In our cohort, these windows generally reflect daytime activity and nighttime rest; however, they are actigraphy data‐driven and not inherently restricted to daytime or nighttime. Finally, (v) their corresponding midpoints (M10midpoint and L5midpoint) serve as phase markers, reflecting whether individuals tend to be more active or inactive earlier or later in the day; (vi) the relative amplitude (RA), calculated as (M10−L5)/(M10+L5), is used as an indicator of rhythm robustness, with range from 0 to 1, where higher values represent greater contrast between daytime and nighttime activity, reflecting stronger circadian organization; and (vii) the inter‐day variability (IDV) index, a novel measure of day‐to‐day variability in activity patterns based on a non‐parametric data‐driven spectral decomposition procedure, calculated as the squared largest singular value of the activity matrix, constructed with intra‐day activities as rows, normalized by the sum of the squared four largest singular values of the matrix. The IDV index quantifies how similar daily activity patterns are across days. If day‐to‐day activity patterns are very similar (less inter‐day variability/more stability), most of the variability is captured by a single component, resulting in a higher IDV index, and if daily patterns differ from each other (higher IDV/less stability), the variability is spread across several components, and the IDV index is lower.

*Parametric circadian RAR* measures calculated through cosinor modeling included (i) midline estimating statistic of rhythm (MESOR), defined as the average activity over the 24‐h period; (ii) amplitude, which represents the difference in magnitude of activity between active and rest phases; (iii) acrophase, indicating the timing of peak activity. For each participant, daily M10, L5, and RA values were calculated and then averaged across all valid actigraphy days to be in subsequent group comparisons and correlation analyses.

Nocturnal sleep parameters were derived using the Cole‐Kripke algorithm in ActiLife software version 6.^32^ The sleep variables analyzed included time in bed (TIB [minutes]), total sleep time (TST [minutes] as the number of minutes slept while in bed), and SE (%, calculated as TST/time in bed ×100). Additional sleep continuity measures included sleep latency (SL [minutes], defined as the time spent awake until the first epoch of sleep and sleep onset), wake after sleep onset (WASO [minutes], representing the total time spent awake after initial sleep onset), and the number of awakenings throughout the night (awakenings).

Additionally, for a subset of participants, polysomnography (PSG) data collected after actigraphy in a separate study were leveraged for the present analyses. Additional analyses included nocturnal sleep architecture and respiratory parameters, including REM sleep duration, slow‐wave sleep (N3) duration, and the apnea–hypopnea index (AHI), defined as the total number of apneas (complete cessation of airflow for ≥10 s) and hypopneas (reductions in airflow lasting ≥10 s accompanied by either cortical arousal or ≥3% oxygen desaturation) per hour of sleep. These measures were selected based on our prior evidence demonstrating significant differences between adults with DS and controls and their potential relevance to AD.^8^, ^11^ Sleep studies were conducted using the Compumedics E Series System with Profusion Sleep Software (Compumedics PSG3, version 3.4). Further methodological details and scoring criteria have been described elsewhere.^11^

### 2.5 Statistical analyses

All statistical analyses were performed with R software version 4.3.3. Differences in sociodemographic and medical characteristics between DS and HC participants were assessed using the *compareGroups* library.^33^ This package tests the normality of continuous variables and subsequently performs parametric or non‐parametric statistical tests accordingly. Chi‐squared tests were used for categorical variables, such as sex and apolipoprotein E (APOE) ε4 status, while Mann‐Whitney tests were applied for continuous variables.

After confirming with the Shapiro‐Wilk test that most of the continuous variables of interest did not follow a normal distribution, we applied general linear models (GLMs) when assessing differences between groups: (i) HC versus DS, (ii) clinical status within DS: aDS versus sDS (considering together pDS and dDS).

For RAR variable analyses, age, sex, TST, SE, REM, and N3 duration and AHI were included as covariables in models for the group comparisons performed between HC and DS, as these PSG sleep features showed between‐group differences and were additionally adjusted for to ensure the results remained robust. Comparisons within the DS group were not adjusted by age because in this population it closely tracks AD pathophysiology and clinical stage, and adjusting for it could lead to overadjustment by removing disease‐related variance. Comparisons within the DS group were not adjusted by age. Post hoc pairwise comparisons following the detection of group differences were performed using Tukey's Honestly Significant Difference (HSD) test. Chi‐squared, or Fisher exact, tests (used when low frequencies were observed) were used for categorical variables such as sex or clinical status. Also, GLMs were applied to evaluate the effect of age and sex on circadian RAR variables adjusting for AHI, SE, or TST (and sex for the age models). Interaction terms were included to determine whether the relationship between age or sex and circadian measures differed between groups (HC vs DS and clinical status within the DS group).

## 3 RESULTS

### 3.1 Participant characteristics

A total of 188 participants were enrolled in the study (147 adults with DS and 41 controls). Seven participants (two asymptomatic DS and five DS with AD dementia) were excluded because their actigraphy recordings were shorter than 36 continuous hours due to limited tolerance of the device. The final analytic sample included 181 individuals: 140 adults with DS (108 aDS, 32 sDS) and 41 HCs. A subset of 118 (17 HC and 101 DS) were screened for APOE haplotype. Table 1 summarizes the demographic characteristics of the study participants. HCs were older than individuals with aDS (*p* < 0.001), whereas no age differences were found between HC and sDS (*p* > 0.05). There were no significant differences in the distribution of sex or in the proportion of APOE ε4 carriers across groups.

**TABLE 1:** Participants’ demographic characteristics and medical data.

Characteristic	HC *N* = 41	DS *N* = 140	aDS *N* = 108	sDS *N* = 32	*p* value HC versus DS	*p* value HC versus aDS	*p* value HC versus sDS	*p* value aDS versus sDS
Age Median [IQR], years	53.00 [43.00; 61.00]	42.00 [33.75; 50.00]	39.00 [32.00; 45.00]	52.00 [50.75; 57.00]	<0.001***	<0.001***	0.785	<0.001***
Sex								
Female, *n* (%)	19 (46.34)	64 (45.71)	46 (42.59)	18 (56.25)	1.000	0.820	0.818	0.738
Intellectual disability								
Mild, *n* (%)		54 (38.57)	46 (42.59)	8 (25.00)				0.069
Moderate, *n* (%)		75 (53.57)	56 (51.85)	19 (59.38)	19 (59.38)			
Severe/profound, *n* (%)		11 (7.86)	6 (5.56)	5 (15.62)	5 (15.62)			
APOE status								
ε4+, *n* (%)	5 (29.41)	18 (17.82)	15 (20.27)	3 (11.11)	0.320	0.516	0.516	0.516
Vascular risk factors								
Hypertension, *n* (%)	3 (13.64)	2 (2.67)	1 (1.82)	1 (5.00)	0.075	0.204	0.608	0.608
Diabetes mellitus, *n* (%)	0 (0.00)	3 (4.00)	2 (3.64)	1 (5.00)	1.000	1.000	1.000	1.000
Dyslipidemia, *n* (%)	6 (27.27)	10 (13.33)	7 (12.73)	3 (15.00)	0.187	0.530	0.689	1.000
Other medical conditions								
Epilepsy, *n* (%)	0 (0.00)	7 (6.86)	2 (2.44)	5 (25.00)	<0.001***	<0.001***	<0.001***	<0.001***
Hypothyroidism, *n* (%)	1 (12.50)	34 (61.82)	25 (56.82)	9 (81.82)	0.018*	0.074	0.016*	0.174
Treatment								
Use of any psychotropic medication, *n* (%)	4 (66.67)	58 (50.88)	38 (46.34)	20 (62.50)	0.681	0.629	1.000	0.440

*Note*: Data are *n* (%) or median [IQR]. Statistical differences with asterisks denoting significance thresholds: ***(*p* < 0.001), **(*p* < 0.01), and *(*p* < 0.05). Note that APOE genotyping was available for a subset of 118 participants (17 HC and 101 DS, including 74 aDS and 27 sDS).

Abbreviations: aDS, asymptomatic Down syndrome; APOE, apolipoprotein E; DS, Down syndrome; HC, euploid cognitively unimpaired healthy controls; IQR, interquartile range; sDS, Down syndrome symptomatic for Alzheimer's disease dementia.

Additionally, there were no significant group differences in the prevalence of hypertension, dyslipidemia, or diabetes mellitus. A substantial proportion of individuals with DS presented with epilepsy (*N* = 7, 6.86%, *p* < 0.001) and hypothyroidism (*N* = 34, 61.82%, *p* < 0.018), both of which became more frequent with AD progression (epilepsy: aDS 2.44% vs sDS 25%, *p* < 0.001; hypothyroidism: aDS 56.82% vs sDS 81.82%, *p* = 0.011).

There were no significant differences in treatment profiles between the DS and HC groups, nor among DS subgroups. Importantly, sleep measures did not differ significantly between individuals with DS receiving psychotropic medication and those not undergoing active treatment (Table S1). Although a significant difference was observed for IS (*p* = 0.017), this was the only circadian metric showing such variation.

### 3.2 Actigraphy‐derived RAR measures

Compared to HCs, adults with DS showed marked alterations in circadian RAR. DS presented significantly higher nighttime activity levels (L5: *p* = 0.001), but not a decreased daytime activity (M10: *p > *0.05) compared to HCs. The rhythm robustness, RA, was significantly reduced in DS compared to HCs (*p *= 0.005), demonstrating a weakened day‐night contrast. Moreover, the IV was significantly lower (*p* = 0.006), and the IS values were significantly higher in DS versus HC (*p* = 0.035), which indicates less circadian rhythm fragmentation and better synchronization of the rhythm. The IDV index was significantly higher in DS than in HCs (*p <* 0.001), indicating greater stability in daily activity profiles. In other words, individuals with DS exhibited less pronounced day‐to‐day fluctuations in activity pattern. Midpoint measures for M10 and L5 did not differ between groups (*p *> 0.05), and the parametric rhythm measures (i.e., MESOR, amplitude, and acrophase) did not show significant group differences (*p *> 0.05). For further details see Figure S1.

**FIGURE 1:**
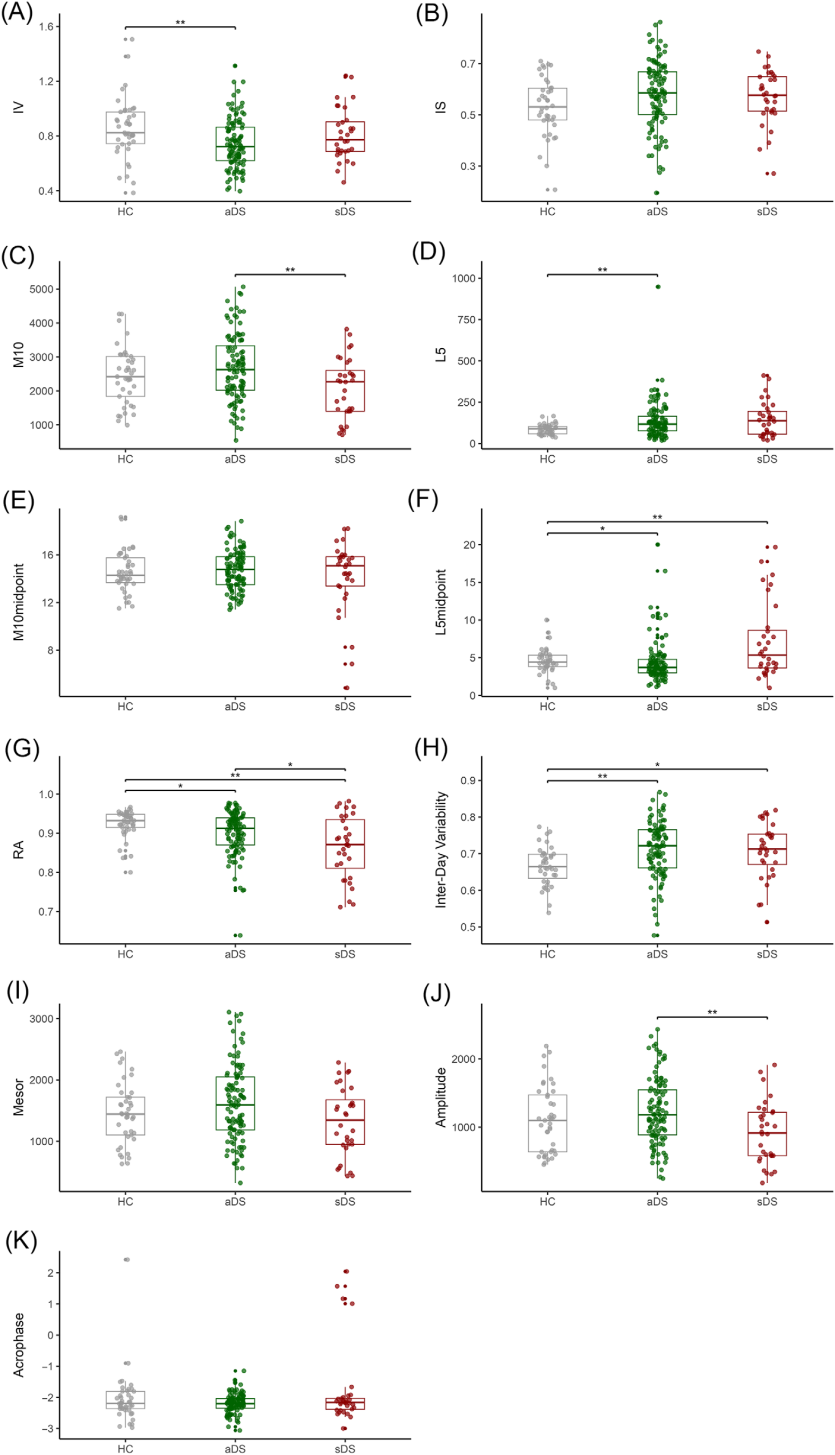
Boxplots for RAR measures by group. Values for (A) IV, (B) IS, (C) M10, (D) L5, (E) M10midpoint, (F) L5midpoint, (G) RA, (H) inter‐day variability, (I) MESOR, (J) amplitude, and (K) acrophase are shown by group of diagnosis. Jittered points display individual data within each group: HC (gray), aDS (green), sDS (red). The asterisk (*) indicates significant values (*p* < 0.05). aDS, asymptomatic Down syndrome; HC, euploid cognitively unimpaired healthy controls; IS, interdaily stability; IV, intradaily variability; M10, activity level of most active 10‐h period; L5, activity level of least active 5‐h period; RAR, rest–activity rhythm; RA, relative amplitude; sDS, symptomatic Down syndrome.

Circadian disruption worsened across the AD continuum within DS. Individuals with DS and symptomatic AD (i.e., sDS) presented with lower daytime activity (M10 aDS vs sDS: *p *= 0.004) than those aDS and higher nighttime activity (L5: *p* = 0.006). RA decreased further in sDS (HC vs sDS: *p* = 0.005; aDS vs sDS: *p* = 0.023), while IV and IS did not present significant differences between AD stages. L5midpoint was significantly higher in dDS compared to aDS (*p *= 0.006), suggesting later timing of the least‐active period. MESOR and amplitude declined from aDS to sDS, with both metrics significantly lower for the symptomatic participants (*p* = 0.027 and *p* = 0.008, respectively), whereas acrophase remained comparable across AD stages. The IDV index did not significantly differ across AD stages, although significantly higher values in both DS groups versus HCs were observed (HC vs aDS: *p *= 0.002; HC vs sDS: *p *= 0.043), indicating early circadian alteration preceding clinical dementia (Figure 1 and Table 2).

**TABLE 2:** Circadian RAR variables.

Variable	HC *N* = 41	DS *N* = 140	aDS *N* = 108	sDS *N* = 32	*p* value HC versus DS	*p* value HC versus aDS	*p* value HC versus sDS	*p* value aDS versus sDS
M10	2419.44 [1835.88; 3014.05]	2475.85 [1856.48; 3171.01]	2626.70 [2017.90; 3331.51]	2269.22 [1400.17; 2602.63]	0.538	0.360	0.216	0.004**
L5	89.58 [59.32; 102.83]	119.33 [68.97; 175.72]	117.60 [76.84; 164.88]	138.07 [56.63; 195.16]	0.001**	0.004**	0.060	0.670
RA	0.93 [0.91; 0.95]	0.90 [0.86; 0.94]	0.91 [0.87; 0.94]	0.87 [0.81; 0.93]	0.005**	0.023*	0.005**	0.023*
M10midpoint	14.29 [13.67; 15.75]	14.83 [13.50; 15.83]	14.77 [13.50; 15.83]	15.08 [13.38; 15.83]	0.510	0.990	0.990	0.990
L5midpoint	4.43 [3.83; 5.33]	4.00 [3.00; 5.33]	3.71 [3.00; 4.79]	5.35 [3.65; 8.62]	0.158	0.027*	0.120	0.006**
IV	0.82 [0.74; 0.98]	0.73 [0.64; 0.88]	0.72 [0.62; 0.86]	0.77 [0.69; 0.90]	0.006**	0.007**	0.276	0.151
IS	0.53 [0.48; 0.60]	0.58 [0.51; 0.67]	0.59 [0.50; 0.67]	0.58 [0.51; 0.65]	0.035*	0.101	0.405	0.924
MESOR	1443.81 [1103.00; 1718.94]	1561.06 [1111.67; 1969.13]	1593.79 [1186.56; 2048.43]	1345.48 [948.14; 1676.71]	0.299	0.260	0.576	0.027*
Amplitude	1098.86 [638.51; 1471.42]	1115.94 [830.90; 1495.74]	1180.67 [888.12; 1546.20]	914.78 [583.51; 1217.30]	0.517	0.158	0.158	0.008**
Acrophase	−2.19 [−2.36; −1.81]	−2.19 [−2.36; −2.04]	−2.20 [−2.35; −2.04]	−2.16 [−2.39; −2.03]	0.740	0.833	0.833	0.833
Inter‐day variability	0.66 [0.63; 0.70]	0.72 [0.66; 0.76]	0.72 [0.66; 0.77]	0.71 [0.67; 0.75]	<0.001***	0.002**	0.043*	0.924

*Note*: Data are median [IQR]. Statistical differences with asterisks denoting significance thresholds: ***(*p* < 0.001), **(*p* < 0.01), and *(*p* < 0.05).

Abbreviations: aDS, asymptomatic Down syndrome; HC, euploid cognitively unimpaired healthy controls; IS, interdaily stability; IV, intradaily variability; M10, activity level of the most active 10‐h period; L5, activity level of the least active 5‐h period; RAR, rest–activity rhythm; RA, relative amplitude; sDS, symptomatic Down syndrome.

### 3.3 Nocturnal sleep actigraphy parameters

Compared to the HC, adults with DS present worse sleep continuity measures, with longer SL (*p* = 0.019) and lower SE (*p* = 0.035). Adults with DS spent more time in bed (TIB: *p* < 0.001) but did not sleep longer, as TST did not differ between groups (*p* > 0.05). WASO was markedly higher in DS than in HC (*p* = 0.002), although the number and duration of awakenings did not significantly differ between groups (*p* > 0.05). Further, sleep measures worsened across the AD continuum, although group differences did not reach statistical significance when aDS and sDS groups were compared (all *p* values > 0.05). For further details see Figure S2.

### 3.4 Relationship between circadian RAR measures and nocturnal sleep architecture

PSG data were available for most participants (121/140 DS; 38/41 HCs) and were obtained based on logistical availability within concurrent sleep‐unit studies rather than clinical characteristics or actigraphy‐derived outcomes. PSG availability was high and comparable across groups. Obtained differences in circadian RAR (higher and delayed nighttime activity and reduced amplitude) persisted after adjusting for sleep duration (i.e., TST), SE, and AHI. To further explore the effect of OSA on the studied RAR variables, we additionally examined differences across OSA severity groups (i.e., no OSA, mild, moderate, and severe), as shown in Figure S3. PSG data show, as expected, a lower percentage of REM sleep (7.30 vs 18.80%; *p* < 0.001), higher in N3 (28.60 vs 21.75%; *p* = 0.002), and higher prevalence of OSA based on AHI (16.95 vs 3.00; *p* < 0.001) compared to HCs (for further details see Table S2). REM sleep and N3 sleep duration showed no consistent associations with most circadian RAR metrics across DS and controls or across the AD continuum. These null associations persisted after adjustment for sex, sleep duration, and OSA severity. Only REM sleep duration (in minutes) showed a significant or near‐significant association with circadian amplitude, such that shorter REM duration was associated with lower rhythm amplitude, with a stronger relationship in aDS than in HCs (*p *= 0.0378).

### 3.5 Age‐related changes for actigraphy data

Age‐related effects on circadian RAR measures did not differ between DS and controls. Specifically, in analyses testing age‐by‐group interactions, age did not significantly interact with group status for any RAR parameter in either the unadjusted models or those adjusted for (i) sex, (ii) sex and AHI, (iii) sex and TST, or (iv) sex and SE. The only exception was acrophase, which initially showed a significant group interaction, indicating that individuals with DS exhibited a slightly more delayed circadian phase with age compared to HCs. However, this effect disappeared after adjustment for sex and AHI or for sex and TST. Across the AD continuum, acrophase demonstrated a significant age‐by‐clinical diagnosis interaction that was specific to the sDS group, with older adults in the dementia stage showing a progressively delayed peak time activity (all *p* < 0.05).

**FIGURE 2:**
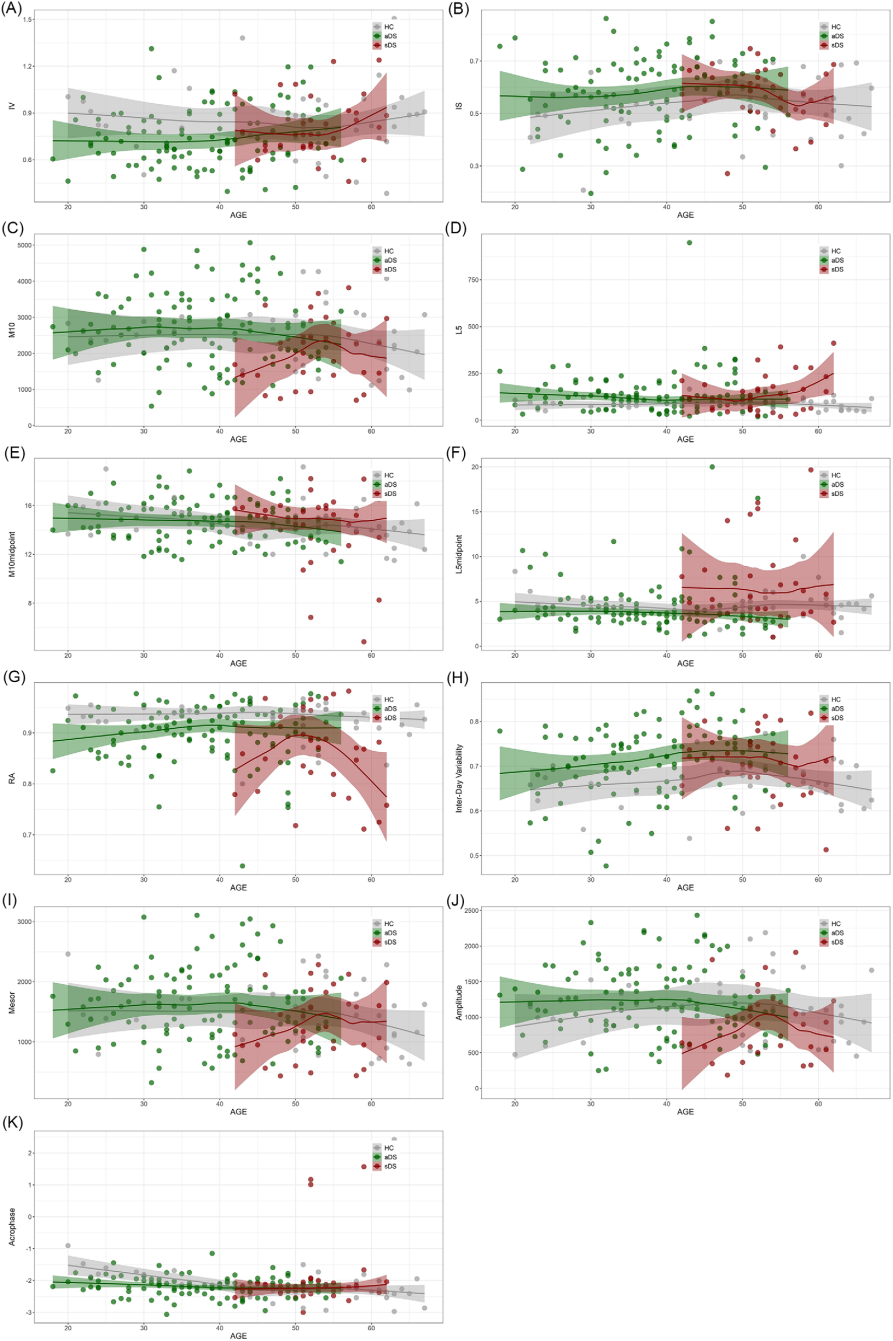
Group interactions across RAR measures and age. Scatter plots showing association between (A) IV, (B) IS, (C) M10, (D) L5, (E) M10midpoint, (F) L5midpoint, (G) RA, (H) inter‐day variability, (I) MESOR, (J) amplitude, and (K) acrophase with age, by group. Individual data points are colored by diagnosis groups: HC (gray), aDS (green), sDS (red), and locally estimated scatterplot smoothing curves with confidence interval shaded lines highlighting overall trend within each group. aDS, asymptomatic Down syndrome; HC, euploid cognitively unimpaired healthy controls; IS, interdaily stability; IV, intradaily variability; M10, activity level of most active 10‐h period; L5, activity level of least active 5‐h period; RAR, rest–activity rhythm; RA, relative amplitude; sDS, symptomatic Down syndrome.

No other age‐related effects were observed for any additional non‐parametric or parametric RAR measures. For further details see Figure 2.

### 3.6 Sex differences on actigraphy data

No statistically significant sex‐related differences in RAR measures were observed in adults with DS or HCs. In nocturnal sleep measures only, DS females presented higher TIB and WASO than euploid females (*p* < 0.05). See Figures S4 and Table S3.

## 4 DISCUSSION

This study provides the first comprehensive characterization of circadian RAR patterns in adults with DS across the AD continuum. Several key findings emerge. First, adults with DS exhibit weaker circadian rhythms characterized by reduced day–night contrast and increased nocturnal activity, and these features are evident even before dementia onset. Second, circadian disruption progressively worsened across AD stages with a further reduction in daytime activity and circadian rhythm amplitude, while rhythm regularity and phase timing remained largely preserved, manifesting abnormalities in symptomatic clinical AD stages. Third, RAR alterations persisted after adjustment for sex, age, sleep efficiency, REM and deep sleep duration, and OSA severity, indicating that intrinsic AD‐related mechanisms rather than sleep disruption alone drive circadian dysregulation in DS. As in the general population, circadian RAR dysfunction in adults with DS emerges early in the AD continuum, with the potential for significant clinical impact.

Our findings closely parallel observations in euploid populations, where reduced relative amplitude (lower circadian strength) and increased nocturnal activity represent some of the earliest circadian alterations associated with AD.^12^, ^13^ Patients with MCI already exhibit a weakened day–night activity contrast, with further deterioration in AD dementia, consistent with a progressive loss of circadian robustness with disease severity.^18^, ^19^, ^34^ Large prospective studies in middle‐age and older adults further demonstrate that lower RA and higher nighttime activity independently predict incident MCI and dementia, greater Aβ and tau burden, and faster cognitive decline.^23^, ^26^ The same graded pattern was observed in our DS cohort: early RA reductions in asymptomatic individuals followed by further deterioration with dementia, further supporting RA as a sensitive marker of disease severity. These findings suggest that RAR alterations track AD progression in DS as they do in sporadic AD. Future multimodal cross‐sectional and longitudinal studies integrating circadian RAR measures with AD biomarkers are needed to determine the causal relationship between circadian rhythms and AD‐related neurodegeneration in this population.

In our study, circadian disruption in adults with DS was mainly driven by weakening of rhythm strength, with relative amplitude, MESOR, and amplitude showing the greatest deterioration across the AD continuum, whereas phase‐based measures of regularity and timing were largely preserved. Phase shifts emerged only at the dementia stage, reflected by a delayed L5 midpoint, suggesting that circadian phase disruption in DS arises primarily with overt neurodegeneration. Similar patterns are described in euploid populations, with diminished rhythm strength and increased fragmentation emerging as early circadian changes, whereas associations between phase‐based metrics and dementia risk are inconsistent and vary by the specific measure and ranging from null^35^ to advanced^24^, ^26^ or delayed.^25^

Unexpectedly, adults with DS exhibited lower rhythm fragmentation (IV) and higher circadian stability than euploid controls, suggesting more stable day‐to‐day rest–activity patterns and well‐synchronized 24‐h routine. This contrasts with aging, MCI, and AD studies in euploid population, where increased fragmentation and reduced regularity are consistently associated with cognitive decline,^18^, ^36^ higher AB burden,^12^, ^25^ reduced cortical thickness,^37^ and faster progression to dementia.^6^, ^19^ However, preserved or even increased circadian regularity has also been described in institutionalized or care‐supported populations, where externally imposed schedules constrain day‐to‐day variability despite underlying neurodegeneration.^38^, ^39^ Phase‐based circadian metrics are particularly sensitive to behavioral and social cues. Our results likely reflect the highly structured daily routines common in DS, such as fixed wake times, scheduled activities, and regular mealtimes determined by caregivers or residential programs, that may mask intrinsic circadian dysregulation. These findings suggest that phase‐based circadian measures may be less sensitive to intrinsic circadian dysfunction in care‐supported populations, highlighting the need for biological timing markers such as melatonin or core body temperature to accurately assess circadian phase.^23^

Importantly, the limited coupling between circadian RAR measures and PSG sleep architecture and respiratory index further supports the likelihood that circadian dysregulation in DS is not simply secondary to reduced sleep duration or OSA but is due to intrinsic neurobiological factors. REM and slow‐wave sleep showed no consistent associations with most RAR metrics, and circadian group differences persisted after adjustment for OSA severity and sleep efficiency. Only rhythm strength measures seemed to have an increasing trajectory with increasing REM minutes, which may reflect parallel manifestations of underlying shared neurodegenerative processes. REM sleep regulation and circadian amplitude depend on the integrity of brainstem–hypothalamic circuitry, including the locus coeruleus (LC) and its projections to the SCN, which are earliest targets of tau pathology in AD.^40^, ^41^ LC dysfunction has been linked to sleep–wake instability and RAR fragmentation,^40^, ^41^, ^42^ and recent neuropathological studies indicate that LC vulnerability occurs earlier and is more severe in DS than in the general population, potentially accelerating the emergence of RAR abnormalities.^43^, ^44^ Beyond the LC, AD‐related tau pathology also targets key wake–sleep and entrainment nodes with direct projections relevant to circadian output, including wake‐promoting orexinergic neurons in the lateral hypothalamus and histaminergic neurons in the tuberomammillary nucleus, as well as in the serotonergic dorsal raphe pathways that provide non‐photic input to the SCN,^22^, ^45^, ^46^ which might contribute to the progressive loss of circadian robustness.

Other biological mechanisms specific to DS likely amplify susceptibility to early circadian disruption. Lifelong APP overexpression accelerates Aβ and tau accumulation, and even subtle preclinical elevations of these biomarkers are associated with reduced circadian amplitude and increased fragmentation in sporadic AD.^12^ This occurs against a background of chromosome 21 gene‐dosage effects that impair oxidative balance, mitochondrial function, and proteostasis, thereby weakening molecular clock function and reducing the resilience of the SCN.^47^ Additional disruption of SCN signaling through Aβ‐mediated clock gene dysfunction and neuroinflammatory processes may further compound these effects.^48^, ^49^ Emerging evidence links sleep–wake–related thermoregulatory fluctuations to extracellular tau dynamics, suggesting a mechanistic pathway through which circadian and temperature dysregulation may contribute to tau‐driven neurodegeneration.^50^ Together, these converging mechanisms support the concept that individuals with DS may reach a pathological threshold capable of destabilizing circadian circuits substantially earlier than euploid individuals, with the potential for bidirectional exacerbation with neurogenerative change.

To our knowledge, only one prior study examined circadian RAR patterns in DS, reporting attenuated rhythmicity from infancy with increasing fragmentation across age.^27^, ^41^ We did not observe generalized age‐related changes in RAR measures. Instead, age‐related phase alterations emerged only in DS individuals with dementia, characterized by delayed acrophase and later nocturnal inactivity, as observed in sporadic AD.^18^ While normal aging is typically associated with circadian phase advance due to weakened SCN output,^51^, ^52^ AD‐related neurodegeneration appears to disrupt circadian timing networks, leading to pathological phase delay.^49^ The delayed timing of nocturnal inactivity indicates increasing nighttime activity and loss of temporal consolidation with dementia progression, mirroring advanced circadian disorganization observed in sporadic AD.^18^, ^41^

We did not observe sex differences in circadian RAR measures in adults with DS. This contrasts with findings in euploid populations, reporting greater fragmentation and weaker circadian robustness in men.^18^, ^23^, ^53^, ^54^ One possible explanation might be that sex‐related behavioral and social roles, such as household responsibilities or caregiving may promote greater circadian regularity in women, but such differences may be attenuated or absent in DS. Biologically, lower melatonin levels in men may weaken circadian regulation and exacerbate tau pathology.^55^ Larger studies that include biological and socio‐behavioral factors are needed to clarify sex‐ and gender‐related differences in circadian dysfunction and their relevance to AD risk in DS.

Our findings highlight the clinical value of assessing RAR patterns in DS. Continuous, non‐invasive monitoring of daily activity using wearable devices provides a feasible approach to detecting circadian disturbances that often remain unrecognized yet may meaningfully impact quality of life. These results also underscore the importance of caregiver education and open avenues for circadian‐targeted interventions, such as light therapy, behavioral scheduling, or melatonin, to improve circadian health and potentially mitigate cognitive decline, as suggested in other AD populations.^46^, ^53^

To our knowledge, this is the largest study to date evaluating circadian RAR in adults with DS and the first to evaluate the impact of AD on circadian RAR across the AD continuum. The strengths of this study include its large, community‐based DS cohort spanning a wide age range, harmonized clinical AD staging, inclusion of a euploid control group and objective circadian assessment using actigraphy with both parametric and non‐parametric analysis, and PSG data. Rigorous adjustment for sex, SE, and OSA severity within a unique human genetic model of AD with sleep disorders allowed the distinction of circadian from sleep‐driven effects.

Our study has several limitations. First, the relatively smaller sample size in the dementia subgroup may have limited detection of subtle effects; nevertheless, symptomatic AD in DS remains relatively rare in longitudinal cohorts due to the young age of most participants symptomatic, and our sample is one of the largest, well‐characterized in this setting. Second, the cross‐sectional design precludes causal inference, underscoring the need for longitudinal studies to determine whether circadian weakening precedes or follows AD biomarker changes. Third, while we adjusted for major confounders, we cannot exclude the potential influence of unmeasured factors such as physical activity or light exposure. Finally, because we relied on actigraphy‐based behavioral rhythms, future work should integrate biological circadian markers (e.g., melatonin, cortisol, and core body temperature) together with AD biomarkers to better delineate mechanistic links between circadian dysregulation and neurodegeneration in DS. Fourth, the IDV index analysis done in this work was exploratory, and further validation of the index is needed.

In summary, adults with DS exhibit early, progressive loss of circadian RAR robustness as a core feature of DS‐associated AD that is not explained by sleep disruption. These findings support RAR alterations as a sensitive early marker of circadian dysfunction and neurodegenerative vulnerability in DS. Further multimodal longitudinal studies are needed to evaluate their role as a modifiable risk factor to slow cognitive decline and modify AD trajectories in this high‐risk population.

## CONFLICT OF INTEREST STATEMENT

J.F. reported receiving personal fees for service on the advisory boards, adjudication committees, or speaker honoraria from AC Immune, Adamed, Alzheon, Biogen, Eisai, Esteve, Fujirebio, Ionis, Laboratorios Carnot, Life Molecular Imaging, Lilly, Lundbeck, Novo Nordisk, Perha, Roche, Zambón and outside the submitted work. D.A., A.L., and J.F. reports holding a patent for markers of synaptopathy in neurodegenerative disease (licensed to ADx, EPI8382175.0). S.G. reported receiving personal fees for service on the advisory boards, speaker honoraria, or educational activities from Esteve, Idorsia Novo Nordisk, and Biojen. M.C.‐I. reported receiving personal fees for service on the advisory boards, speaker honoraria, or educational activities from Esteve, Lilly, Neuraxpharm, Adium, and Roch. J.A. reported receiving personal fees for service on speaker honoraria or educational activities from Lilly, Esteve, Fujirebio‐Europe, and Roche diagnostics. D.A. reported receiving personal fees for advisory board services and/or speaker honoraria from Fujirebio‐Europe, Roche, Nutricia, Krka Farmacéutica, Lilly, Zambon S.A.U., Grifols, and Esteve, outside the submitted work. M.A. reported receiving speaker honoraria from Esteve Pharmaceuticals, Lilly, Neuraxpharm, Kern Pharma, Novo Nordisk, Zambon, and NutriciaAL has served as a consultant or on advisory boards for Almirall, Fujirebio‐Europe, Roche, Biogen, Grifols, Novartis, Eisai, Lilly, and Nutricia, outside the submitted work. The other authors declared no potential conflicts of interest. Author disclosures are available in the Supporting Information.

## CONSENT STATEMENT

All participants and/or their legally authorized representatives gave written informed consent.

## Supporting information



Supporting Information

Supporting Information
