# Supplementary material for: Characterizing circadian rest–activity rhythm patterns across Alzheimer's disease continuum in Down syndrome
Source: Alzheimers Dement. 2026 Apr 29;22(5):e71409. doi: 10.1002/alz.71409 (PMC13128343; doi:10.1002/alz.71409)
Supplement: Supplementary file 2 — Supporting Information [file ALZ-22-e71409-s002.docx]

**SUPPLEMENTARY MATERIAL FOR:**

**Characterizing Circadian Rest–Activity Rhythm Patterns Across Alzheimer’s Disease Continuum in Down Syndrome**

Sandra Giménez^1,2,3,4^ Lídia Vaqué-Alcázar^2,5^, Susana Clos^2^, Bessy Benejam^2,6^, Maria Carmona-Iragui^2,3,6^, Lucía Maure-Blesa^2^, Laura Videla^2,3,6^, Nuole Zhu^2^, Miren Altuna^7,8^ Javier Arranz^2^ , Isabel Barroeta^2,3^, Íñigo Rodríguez-Baz^2,3^, Alexandre Bejanin^2,3^, Ana Bueno^2^, Susana Fernandez^6^, Laura del Hoyo Soriano^2,3^ , Lucia Pertierra^2,9^ , Daniel Alcolea^2,3^, Bruce Miller^4,10^, Lea T Grinberg ^4,11^ , Joaquin Ruiz^12^, Christos Panagiotis Lisgaras^12,13^ , Hau-Tieng Wu^14^, Alberto Lleó^2,3^ , Ricardo S Osorio^12,13^, Esther M. Blessing^12,13*^, Juan Fortea^2,3,6*^.



**Figure S1.** Boxplots for RAR measures by group (HC vs. DS). Values for (A) IV, (B) IS, (C) M10, (D) L5, (E) M10midpoint, (F) L5midpoint, (G) RA, (H) Inter-Day Variability, (I) Mesor, (J) Amplitude, (K) Acrophase are shown by group of diagnosis. Jittered points display individual data within each group: HC (gray), DS (yellow). Abbreviations: aDS, asymptomatic Down syndrome; HC, euploid cognitively unimpaired healthy controls; IS, interdaily stability; IV, intradaily variability; M10, activity level of the most active 10-hour period; L5, activity level of the least active 5-hour period; RAR, rest-activity rhythm; RA, relative amplitude; sDS, symptomatic Down syndrome.


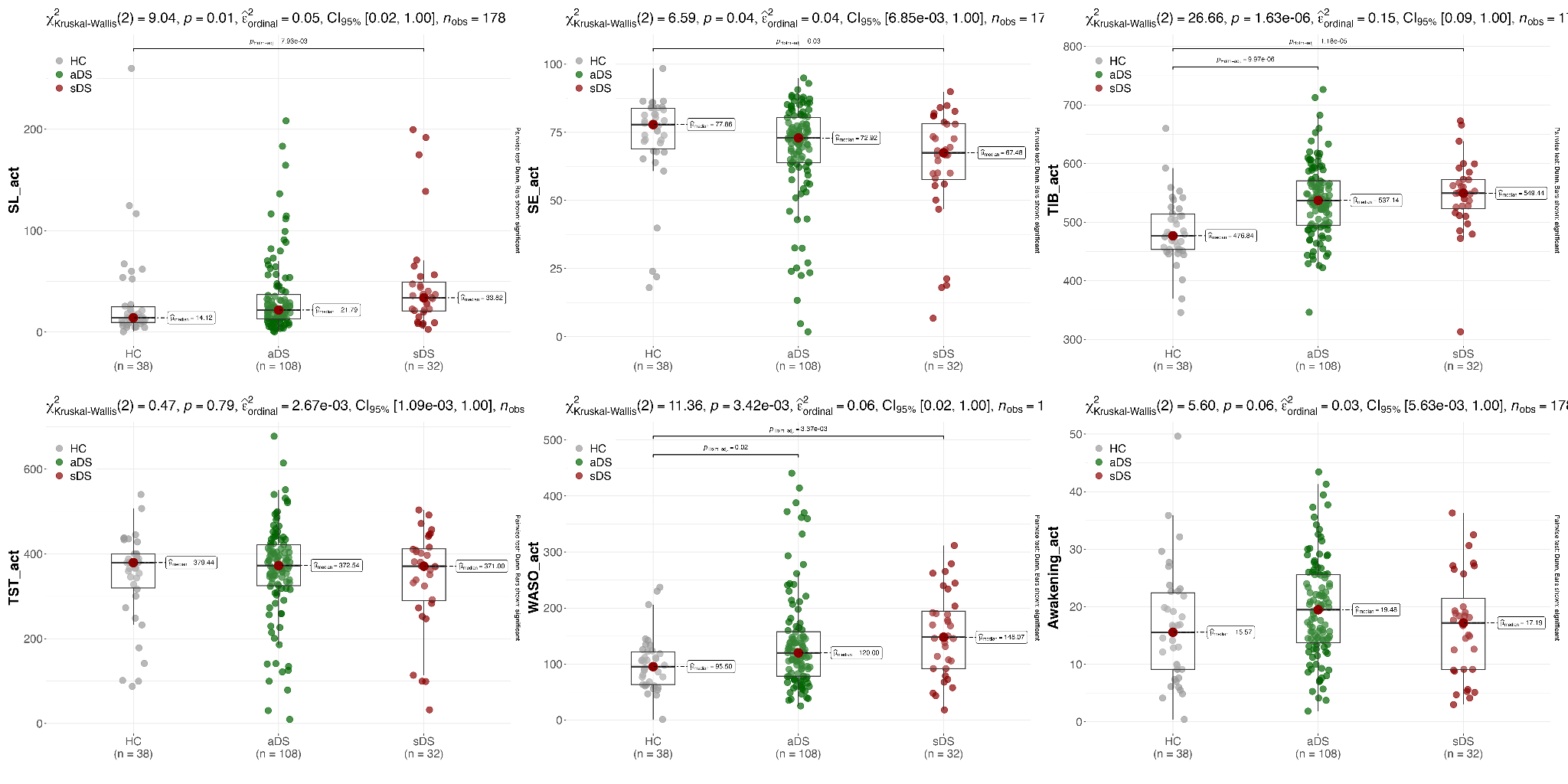


**Figure S2.** Boxplots for nocturnal sleep actigraphy parameters by group. Jittered points display individual data within each group: HC (gray), aDS (green), sDS (red). The star (*) indicates significant values (p<.05). Abbreviations: aDS, asymptomatic Down syndrome; HC, euploid cognitively unimpaired healthy controls; sDS, symptomatic Down syndrome; SE, sleep efficiency; SL, sleep latency; TIB, time in bed; TST, total sleep time; WASO, wake after sleep onset. Note that the suffix ‘_act’ indicates that the corresponding variables represent raw actigraphy-derived parameters.


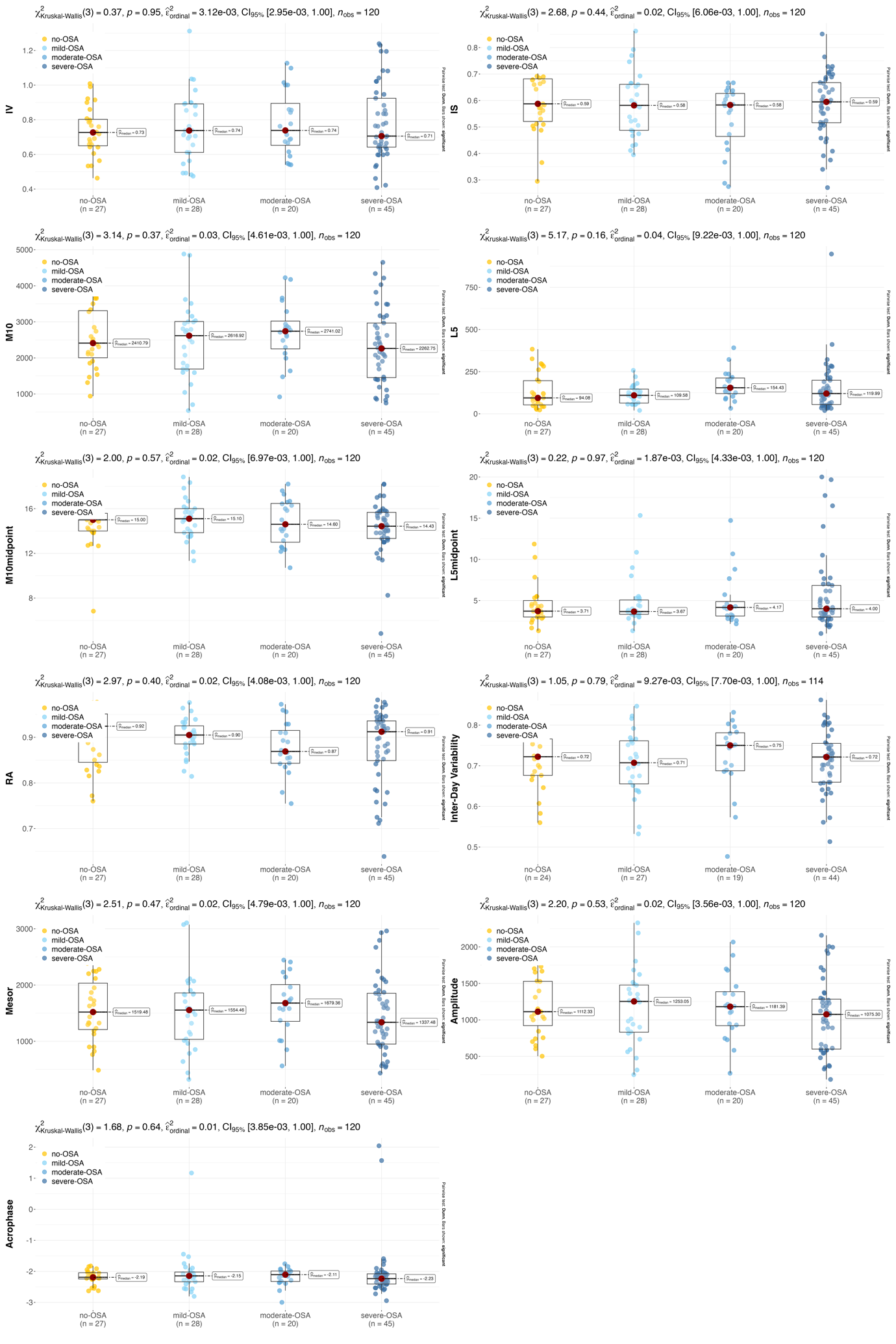


**Figure S3.** Boxplots for RAR measures by OSA. Values for (A) IV, (B) IS, (C) M10, (D) L5, (E) M10midpoint, (F) L5midpoint, (G) RA, (H) Inter-Day Variability, (I) Mesor, (J) Amplitude, (K) Acrophase are shown by group of OSA severity. Jittered points display individual data within each OSA group: IS, interdaily stability; IV, intradaily variability; M10, activity level of the most active 10-hour period; L5, activity level of the least active 5-hour period; RAR, rest-activity rhythm; RA, relative amplitude.



**Figure S4.** Boxplots for RAR measures by sex. Values for (A) IV, (B) IS, (C) M10, (D) L5, (E) M10midpoint, (F) L5midpoint, (G) RA, (H) Inter-Day Variability, (I) Mesor, (J) Amplitude, (K) Acrophase are shown by group of diagnosis. Jittered points display individual data within each sex group: Male (green) and Female (purple), split by diagnostic group: HC (light) and DS (dark). The star (*) indicates significant values (p<.05). Abbreviations: aDS, asymptomatic Down syndrome; HC, euploid cognitively unimpaired healthy controls; IS, interdaily stability; IV, intradaily variability; M10, activity level of the most active 10-hour period; L5, activity level of the least active 5-hour period; RAR, rest-activity rhythm; RA, relative amplitude; sDS, symptomatic Down syndrome.

**Table S1. RAR differences between Non-treated and Treated DS participants.**

|  | **Untreated**  **N=56** | **Treated**  **N=58** | **p-value** |
| --- | --- | --- | --- |
| **M10** | 2552.35  [2054.40;3332.34] | 2290.94  [1491.59;3116.55] | 0.136 |
| **L5** | 118.39  [62.52;199.55] | 117.42  [69.96;155.88] | 0.786 |
| **RA** | 0.91  [0.87;0.93] | 0.90  [0.85;0.94] | 0.537 |
| **M10midpoint** | 15.00  [13.83;16.00] | 14.69  [13.50;15.57] | 0.316 |
| **L5midpoint** | 3.92  [2.96;5.38] | 3.83  [3.15;5.30] | 0.747 |
| **IV** | 0.74  [0.64;0.90] | 0.73  [0.64;0.83] | 0.426 |
| **IS** | 0.57  [0.47;0.63] | 0.60  [0.53;0.67] | 0.017 * |
| **Mesor** | 1590.81  [1174.96;2032.37] | 1432.53  [951.05;1834.33] | 0.207 |
| **Amplitude** | 1149.40  [895.68;1448.99] | 1113.94  [661.89;1470.42] | 0.280 |
| **Acrophase** | -2.17  [-2.33;-1.96] | -2.19  [-2.38;-2.08] | 0.248 |
| **Inter-Day Variability** | 0.70  [0.65;0.75] | 0.73  [0.68;0.78] | 0.075 |

*Note:* Data are median [IQR]. Statistical differences with asterisks denoting significance thresholds: ***(p<0.001), **(p<0.01), and *(p<0.05). Participants with DS were classified as “Treated” if they were receiving any psychotropic medication at the time of assessment. Abbreviations: IS, interdaily stability; IV, intradaily variability; M10, activity level of the most active 10-hour period; L5 ,activity level of the least active 5-hour period; RAR, rest-activity rhythm; RA, relative amplitude.

**Table S2. NPSG data.**

|  | **HC**  **N=38** | **DS**  **N=121** | **aDS**  **N=90** | **sDS**  **N=31** | **p-value**  **HC vs DS** | **p-value HC vs aDS** | **p-value HC vs sDS** | **p-value aDS vs sDS** |
| --- | --- | --- | --- | --- | --- | --- | --- | --- |
| **TST (min)** | 378.00 [352.12; 409.00] | 323.50 [248.50; 380.50] | 333.75 [248.62; 398.50] | 297.00 [243.50; 355.25] | 0.001 ** | 0.013 * | <0.001 *** | 0.175 |
| **SE (%)** | 83.40 [76.60; 88.65] | 68.90 [53.20; 82.00] | 70.35 [51.17; 85.18] | 63.20 [54.55; 77.10] | <0.001 *** | 0.003 ** | <0.001 *** | 0.215 |
| **WASO** | 155.25 [93.62;201.12] | 175.00 [113.00;222.50] | 172.00 [112.25;222.00] | 176.00 [115.75;221.00] | 0.172 | 0.393 | 0.393 | 0.969 |
| **SL (min)** | 21.50 [12.62; 31.50] | 27.00 [15.00; 45.50] | 27.00 [15.00; 43.25] | 27.00 [11.50; 64.75] | 0.091 | 0.301 | 0.321 | 0.974 |
| **TIB (min)** | 476.90 [453.65; 480.98] | 481.00 [463.10; 486.00] | 482.00 [466.63; 487.88] | 474.10 [460.65; 484.25] | 0.002 ** | 0.001 ** | 0.203 | 0.149 |
| **AHI** | 3.00 [0.30; 6.70] | 16.95 [6.75; 42.65] | 15.20 [4.48; 37.65] | 31.95 [10.97; 53.58] | <0.001 *** | <0.001 *** | <0.001 *** | 0.043 * |
| **AHI REM** | 3.30 [0.70; 11.50] | 12.10 [0.00; 37.68] | 11.50 [0.00; 34.25] | 12.40 [1.25; 39.40] | 0.028 * | 0.097 | 0.067 | 0.387 |
| **AHI NREM** | 1.60 [0.20; 6.80] | 17.00 [5.20; 43.55] | 15.00 [4.03; 38.48] | 31.90 [11.52; 51.10] | <0.001 *** | <0.001 *** | <0.001 *** | 0.039 * |
| **NREM N1 (min)** | 27.00 [16.62; 39.25] | 19.00 [11.50; 34.00] | 19.25 [11.62; 35.62] | 18.50 [11.00; 27.25] | 0.018 * | 0.062 | 0.051 | 0.533 |
| **NREM N1 (%)** | 7.50 [4.55;11.03] | 6.40 [3.50;11.60] | 6.05 [3.62;11.50] | 6.80 [3.20;11.55] | 0.0265 | 0.726 | 0.726 | 0.898 |
| **NREM N2 (min)** | 186.00 [149.62; 209.62] | 173.00 [112.00; 220.50] | 170.75 [110.50; 219.62] | 176.00 [115.75; 221.00] | 0.318 | 0.856 | 0.856 | 0.880 |
| **NREM N2 (%)** | 50.90 [47.75;54.82] | 53.90 [46.00;61.70] | 53.15 [46.00;60.03] | 59.20 [45.10;63.80] | 0.107 | 0.240 | 0.240 | 0.397 |
| **NREM N3 (min)** | 87.00 [59.62; 104.75] | 96.00 [49.50; 119.00] | 99.75 [52.50; 121.00] | 78.50 [42.75; 105.50] | 0.431 | 0.286 | 0.499 | 0.286 |
| **NREM N3 (%)** | 21.75 [18.30;27.52] | 28.60 [20.70;38.00] | 28.65 [22.52;38.00] | 27.70 [13.80;33.45] | 0.002 ** | 0.003 ** | 0.217 | 0.381 |
| **REM**  **(min)** | 68.50 [49.88; 84.12] | 25.50 [8.88; 44.00] | 27.00 [9.50; 44.50] | 16.50 [3.00; 29.00] | <0.001 *** | <0.001  *** | <0.001  *** | 0.082 |
| **REM**  **(%)** | 18.80 [15.88;23.73] | 7.30 [3.30;12.20] | 8.15 [3.42;12.38] | 6.00 [2.10;8.45] | <0.001 *** | <0.001 *** | <0.001 *** | 0.129 |
| **ODI** | 0.95 [0.12; 6.68] | 14.10 [3.40; 36.58] | 12.40 [2.35; 30.05] | 20.25 [6.78; 43.27] | <0.001 *** | <0.001 *** | <0.001 *** | 0.085 |
| **T90** | 0.20 [0.00; 1.40] | 2.40 [0.30; 17.00] | 1.45 [0.23; 7.97] | 10.40 [1.25; 19.82] | <0.001 *** | 0.001 ** | <0.001 *** | 0.013  * |
| **AI** | 15.00 [8.80; 27.30] | 22.30 [13.60; 36.60] | 20.40 [13.50; 34.58] | 31.20 [15.65; 38.65] | 0.077 | 0.185 | 0.079 | 0.173 |

*Note:* Data are median [IQR]. Statistical differences with asterisks denoting significance thresholds: ***(p<0.001), **(p<0.01), and *(p<0.05). Abbreviations: aDS, asymptomatic Down syndrome; AHI, apnea–hypopnea index; AHI REM, apnea–hypopnea index during rapid eye movement phase; AHI NREM, apnea–hypopnea index during no-rapid eye movement phase; AI, arousal index; DS, Down syndrome; h, hours; HC, euploid cognitively unimpaired healthy controls; min, minutes; NREM N1, NREM N2, NREM N3, no-rapid eye movement stages N1, N2, and N3; ODI, oxygen desaturation index; sDS, Down syndrome symptomatic for Alzheimer's disease dementia; SE, sleep efficiency; SL, sleep latency; SpO2, basal oxygen saturation; T90, percentage of oxygen saturation < 90% for more than 30% of the sleep registration period; TIB, time in bed; TST, total sleep time; WASO, wakefulness after sleep onset.

**Table S3. RAW actigraphy data by sex and diagnostic group.**

|  | **HC Male**  **N=21** | **HC Female**  **N=17** | **DS Male**  **N=76** | **DS Female**  **N=64** | **p-value** | **p-value  HC: Male vs. Female** | **p-value**  **Male:**  **HC vs. DS** | **p-value**  **DS: Male vs. Female** | **p-value**  **Female:**  **HC vs. DS** |
| --- | --- | --- | --- | --- | --- | --- | --- | --- | --- |
| **SL_act** | 14.00 [10.44; 53.86] | 14.25 [9.29; 22.75] | 21.57 [13.86; 32.05] | 29.75 [14.22; 63.22] | 0.034 | 0.747 | 0.321 | 0.135 | 0.084 |
| **SE_act** | 75.55 [67.70; 83.95] | 78.34 [72.28; 83.27] | 72.75 [63.87; 78.57] | 70.87 [60.71; 80.50] | 0.133 | 0.545 | 0.545 | 0.685 | 0.101 |
| **TIB_act** | 479.29 [453.44;514.71] | 527.80 [494.40;566.70] | 468.57 [453.63;510.14] | 550.54 [526.04;590.72] | <0.001 *** | 0.901 | 0.050 | 0.151 | <0.001  *** |
| **TST_act** | 379.44 [273.00; 400.00] | 366.67 [327.88; 399.89] | 367.37 [314.28; 413.89] | 380.47 [329.42; 435.65] | 0.808 | 0.909 | 0.909 | 0.909 | 0.909 |
| **WASO_act** | 106.00 [63.56; 125.88] | 86.00 [61.22; 109.63] | 120.78 [87.64; 175.26] | 126.85 [81.79; 170.65] | 0.012 * | 0.230 | 0.203 | 0.820 | 0.013 |
| **Awakening_act** | 16.57 [7.63; 21.86] | 14.57 [9.13; 23.13] | 19.48 [14.07; 26.00] | 17.76 [9.96; 22.25] | 0.120 | 0.754 | 0.237 | 0.325 | 0.754 |

*Note:* Data are median [IQR]. Statistical differences with asterisks denoting significance thresholds: ***(p<0.001), **(p<0.01), and *(p<0.05). Abbreviations: HC, euploid cognitively unimpaired healthy controls; DS, Down syndrome; SE, sleep efficiency; SL, sleep latency; TIB, time in bed; TST, total sleep time; WASO, wake after sleep onset. Note that the suffix ‘_act’ indicates that the corresponding variables represent raw actigraphy-derived parameters.
